# Estimating time-varying exposure-outcome associations using case-control data: logistic and case-cohort analyses

**DOI:** 10.1186/s12874-015-0104-0

**Published:** 2016-01-05

**Authors:** Ruth H. Keogh, Punam Mangtani, Laura Rodrigues, Patrick Nguipdop Djomo

**Affiliations:** Department of Medical Statistics, Faculty of Epidemiology and Population Health, London School of Hygiene and Tropical Medicine, Keppel Street, London, WC1E 7HT UK; Department of Infectious Disease Epidemiology, Faculty of Epidemiology and Population Health, London School of Hygiene and Tropical Medicine, Keppel Street, London, WC1E 7HT UK

**Keywords:** Case-control study, Case-cohort study, Cox proportional hazards model, Logistic regression, Time-varying association, Vaccine efficacy

## Abstract

**Background:**

Traditional analyses of standard case-control studies using logistic regression do not allow estimation of time-varying associations between exposures and the outcome. We present two approaches which allow this. The motivation is a study of vaccine efficacy as a function of time since vaccination.

**Methods:**

Our first approach is to estimate time-varying exposure-outcome associations by fitting a series of logistic regressions within successive time periods, reusing controls across periods. Our second approach treats the case-control sample as a case-cohort study, with the controls forming the subcohort. In the case-cohort analysis, controls contribute information at all times they are at risk. Extensions allow left truncation, frequency matching and, using the case-cohort analysis, time-varying exposures. Simulations are used to investigate the methods.

**Results:**

The simulation results show that both methods give correct estimates of time-varying effects of exposures using standard case-control data. Using the logistic approach there are efficiency gains by reusing controls over time and care should be taken over the definition of controls within time periods. However, using the case-cohort analysis there is no ambiguity over the definition of controls.

The performance of the two analyses is very similar when controls are used most efficiently under the logistic approach.

**Conclusions:**

Using our methods, case-control studies can be used to estimate time-varying exposure-outcome associations where they may not previously have been considered. The case-cohort analysis has several advantages, including that it allows estimation of time-varying associations as a continuous function of time, while the logistic regression approach is restricted to assuming a step function form for the time-varying association.

## Background

Case-control studies are widely used to study associations between exposures and disease (or other) outcomes, especially when the outcome is rare. For overviews see Breslow and Day (1980) [1], Breslow (1996) [2] and Keogh and Cox (2014) [3]. In a ‘standard’ case-control study cases are individuals who experienced the outcome of interest within a specified time period and controls are chosen to represent the non-cases in the same population.

In this paper we describe methods for estimating time-varying associations between exposures and outcomes using standard case-control study data, focusing on unmatched and frequency matched studies. Conventional analyses of case-control data using logistic regression do not accommodate time-varying associations. We outline two approaches. One is to estimate associations (odds ratios (OR)) separately within a series of time periods using logistic regression. The second treats the case-control sample as a case-cohort study, with the controls forming the ‘subcohort’. The case-cohort design [4] is a method for selecting a case-control-type sample from a prospective cohort, enabling estimation of hazard ratios (HR) without obtaining complete information for the full cohort. See Onland-Moret et al. (2007) [5] for an overview.

We describe a motivating study before outlining the two proposed approaches and presenting results from a simulation study.

The motivation for this work was a case–control study of the long-term efficacy of infant-BCG (Bacillus Calmette-Guérin) vaccination against tuberculosis (TB), in particular of whether the vaccine efficacy becomes weaker over time since vaccination. Incident cases aged 0 to 19 at the first disease episode were identified retrospectively from those occurring over a 10-year period and recruited to the study. Controls were selected at the same time at which cases were retrospectively identified and chosen to represent the underlying population by sampling households, and so as to obtain approximately equal numbers of cases and controls within a series of birth cohorts.

The vaccination policy in the underlying population recommends administration of BCG before age 1. Participants’ vaccination status was ascertained using a combination of vaccination records, reported history, and inspection for BCG vaccination scar. It was of interest to estimate vaccine efficacy within a series of time periods post-vaccination, and to model the vaccine efficacy smoothly with time since vaccination.

Rodrigues and Smith (1999) [6] give an overview of the use of case-control studies to study vaccine efficacy.

## Methods

We outline two approaches to estimating time-varying exposure-outcome associations using unmatched case-control data:

(i) Performing separate logistic regressions within a series of time periods.
(ii) Treating the study as a case-cohort study and applying a case-cohort analysis.

Both approaches assume that the cases are rare in the underlying population.

We consider a case-control sample containing *n* individuals. The main exposure is denoted *x*, whose association with the outcome may vary over time. A vector of covariates is denoted *z*, which are assumed to have non-time-varying associations with the outcome.

### Logistic regression analysis

We focus on estimating the association between the exposure and the outcome within *L* consecutive non-overlapping time periods, that is assuming a step function form for the time-varying association. A logistic model for the probability of being a case in time period *l* is

1 $$ \log \frac{\text{Pr}\left(D_{l}=1|x,z\right)}{1-\text{Pr}\left(D_{l}=1|x,z\right)}=\delta_{0l}+\delta_{Xl}x+\delta^{T}_{Zl}z $$

where *D*_*l*_ denotes case (*D*_*l*_ = 1) or control (*D*_*l*_ = 0) status in time period *l*, *δ*_*Xl*_ is the log OR for the exposure *x* in time period *l*, and *δ*_*Zl*_ is a vector of log ORs for the covariates *z* in time period *l*. The probabilities Pr(*D*_*l*_ = 1|*x*, *z*) are conditional on the case-control sampling scheme and the intercepts *δ*_0*l*_ do not have a useful interpretation [7]. We now discuss the definition of a case and a control in time period *l*, before outlining the analysis based on model (1).

We define an ‘index time’ for each individual. For cases the index time is the time they became a case, on the relevant time scale, e.g. the age at disease diagnosis. For controls the index time is the time up to which it is known they have not had the event; in the motivating example this was the time of being interviewed for the study. For cases, *D*_*l*_ = 1 if the index time was in time period *l*. The question arises as to how to define controls in period *l*. We propose that a control individual can serve as a control in any time period up to and including that in which their index time falls. Therefore controls can contribute to the analysis in more than one time period. For example, in the motivating example the time scale is age and we assume for now that vaccination occurs at birth. We may wish to estimate the vaccine efficacy in age groups (or equivalently years since vaccination periods) 0–4, 5–9, 10–14, 15–19. Individuals interviewed as controls up to and including age 4 can only appear as controls for cases occurring in the 0–4 age group, while an individual interviewed as a control at age 14, say, may serve as a control in three age groups: 0–4, 5–9, 10–14. Another possibility would be to use control individuals in only one time period. However, this would be inefficient in comparison with our proposed scheme for the reuse of controls across multiple time periods. In the simulation study we investigate alternative control definitions. These issues are connected to the work of Lubin and Gail (1984) [8] and Robins et al. (1986) [9], who discuss control selection in nested case-control studies. We do not allow cases occurring in a given time period to contribute to the analysis as a ‘control’ at any time prior to that at which they become a case.

We let *x*_*i*_ and *z*_*i*_ denote the exposure and covariates respectively for individual *i* (*i* = 1, …, *n*). The full likelihood under the analysis approach proposed above, in which controls are reused across multiple time periods, is

2 $$ {\prod}_{i=1}^n{\prod}_{l=1}^L{\left\{\frac{ \exp \left({\delta}_{0l}+{\delta}_{Xl}{x}_i+{\delta}_{Zl}^T{z}_i\right)}{1+ \exp \left({\delta}_{0l}+{\delta}_{Xl}{x}_i+{\delta}_{Zl}^T{z}_i\right)}\right\}}^{I_{li}\times {D}_{li}}{\left\{\frac{1}{1+ \exp \left({\delta}_{0l}+{\delta}_{Xl}{x}_i+{\delta}_{Zl}^T{z}_i\right)}\right\}}^{I_{li}\times \left(1-{\boldsymbol{D}}_{\boldsymbol{li}}\right)} $$

where *D*_*li*_ takes value 1 for a cases occurring in time period *l* and 0 for individuals eligible to be used as a control in time period *l* according to our proposed criteria. *I*_*li*_ is an indicator of whether individual *i* contributes to the analysis in time period *l*, therefore taking value 0 for control individuals with index time less than the lower limit of period *l* and 1 for a controls with index time greater than the lower limit of period *l*. For cases, *I*_*li*_ is 1 if the case occurs in period *l* and 0 otherwise. In practical terms, for the analysis the data can be arranged so that each case has exactly one row of data and each control has one or more rows of data; one row for each time period up to and including that in which their index time falls. The analysis can be performed in standard software for logistic regression by using interactions between time period and the exposure and covariates, allowing a separate intercept for each time period.

It may be reasonable to assume that the associations between the covariates *z* and the outcome is the same across time periods (*δ*_*Zl*_ = *δ*_*Z*_, for all *l* = 1, …, *L*), or that the intercept is the same over time (*δ*_0*l*_ = *δ*_0_, for all *l* = 1, …, *L*). If common parameters are used across time periods then the use of some individuals as controls within multiple time periods induces dependence between contributions to the likelihood and robust variance estimates should be used.

In the analysis proposed above, we do not allow cases to serve as controls in time periods before which they became a case, as this would result in over-representation of future cases in the control set in a given time period. The controls in a given time period are in fact individuals who remained free of becoming a case up to their index time. Therefore there is technically an under-representation of future cases in the control group in each period. However, when cases are rare in the underlying population we expect this to result in negligible bias.

### Case-cohort analysis

The logistic analysis estimates the exposure-outcome association (an OR) separately within time periods, i.e. assuming a step function, but does not extend to allow estimation of a smooth association over time. The way in which controls are used across time periods is also not ideal in that events happen in continuous time, but controls must be assigned within discrete time periods. The logistic analysis could in theory be performed using a large number of short time periods, to build up a detailed picture of how the exposure-outcome association changes over time. However, in practice the number of time periods that can reasonably be used is restricted by sample size.

**Fig. 1:**
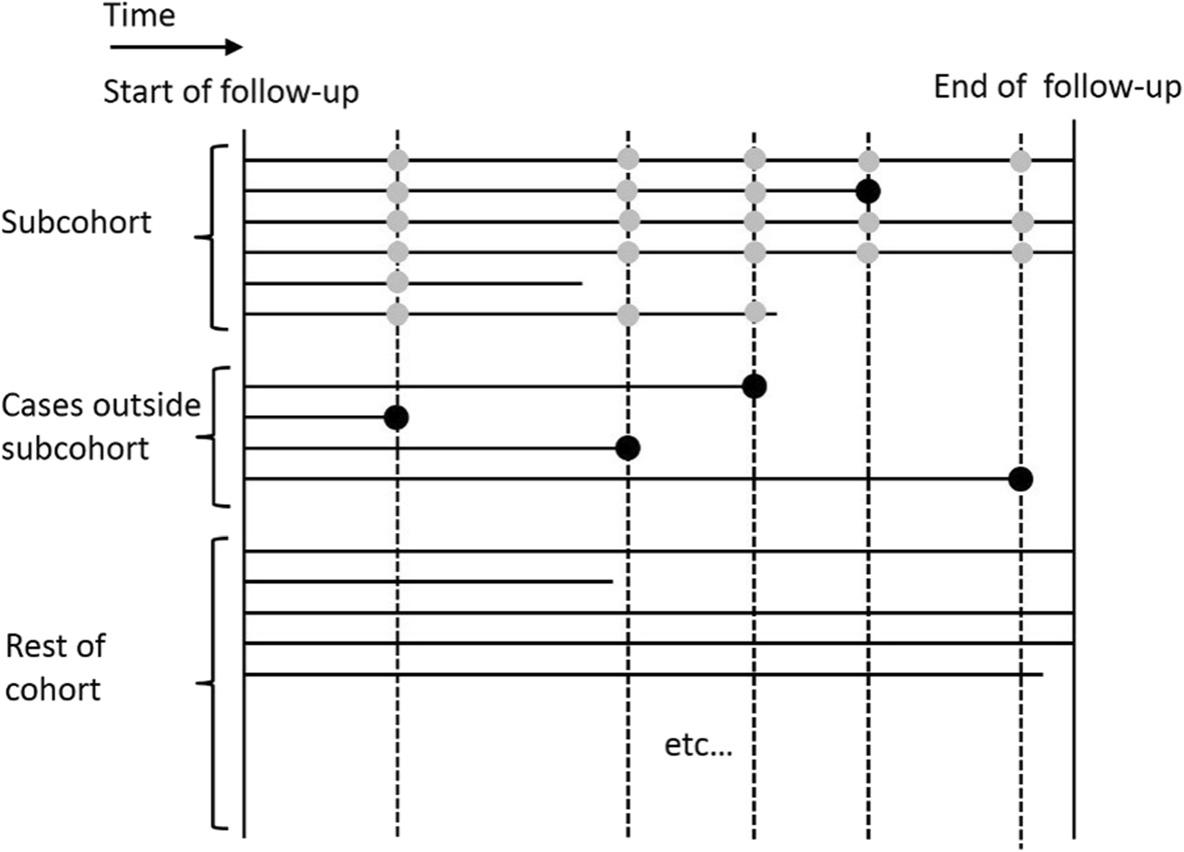
Diagram illustrating a case-cohort study showing survival times (black circles) and censoring times (lines not ending in a circle) in a prospective cohort, also showing the subcohort and the controls used for each case in the case-cohort analysis (grey circles). The full risk set for each case is indicated by the dotted lines

We instead consider a case-cohort analysis and start by describing the standard setting in which a case-cohort study arises as a sub-study within a prospective cohort. To obtain a case-cohort sample the first step is to obtain a random sample of individuals from an underlying cohort at the start of follow-up (or, often, retrospectively, but as though it has been done at the start of follow-up), referred to as the subcohort. The subcohort may contain some individuals who become cases during the course of follow-up. The case-cohort sample is comprised of the subcohort plus all individuals in the rest of the cohort who become cases during the course of follow-up. In the analysis of a case-cohort study each case is compared at its event time with the individuals in the subcohort who are still at risk at that time using a pseudo-partial likelihood (Fig. 1) [7].

In a standard case-cohort analysis, we assume the Cox proportional hazards model [10] for the hazard for the event of interest

3 $$ h\left(t|x,z\right)={h}_0(t) \exp \left(\beta x+{\gamma}^Tz\right) $$

where *t *denotes the event time, *h*_0_(*t*) is the baseline hazard at time *t*, *β* is the log HR for the exposure *x*, and *γ* is a vector of log HRs for the covariates *z*. This can be extended to accommodate a time-varying association between *x* and the hazard, by replacing *β* in (3) by *β*(*t*), which models the log HR for the exposure *x* as a function of time. There are various possibilities for the choice of *β*(*t*). A simple approach is to assume a step function form so that that the HR is assumed constant within a series of time intervals: *β*(*t*) = *v*_1_*β*_1_ + *v*_2_*β*_2_ + ⋯ + *v*_*L*_*β*_*L*_, where *v*_*l*_ is an indicator taking value 1 when *t* is in time period *l* and value 0 otherwise (*l* = 1, …, *L*). Alternatively we can model the exposure-outcome association smoothly as a function of time, for example using a linear model, *β*(*t*) = *β*_0_ + *β*_1_*t*. Another possibility is to use a spline [11]. Quantin et al. (1999) [12] discuss methods for modelling time-varying associations in Cox regression.

We denote the ordered event times *t*_1_ < *t*_2_ < … *t*_*N*_ and the case at time *t*_*j*_ is denoted *i*_*j*_. The parameters of the extended Cox proportional hazards model including *β*(*t*) are estimated using the pseudo-partial likelihood:

4 $$ \prod_{j=1}^N\frac{ \exp \left(\beta \left({t}_j\right){x}_{i_j}+{\gamma}^T{z}_{i_j}\right)}{{\displaystyle {\sum}_{k\in {R}_j}} \exp \left(\beta \left({t}_j\right){x}_k+{\gamma}^T{z}_k\right)} $$

where *R*_*j*_ denotes the set of individuals in the subcohort who were at risk at time *t*_*j*_ (including the case itself at time *t*_*j*_ if the case is in the subcohort), plus the case itself at *t*_*j*_ (if the case is not in the subcohort)[6]. This differs from the partial likelihood analysis of a full cohort study [13] only by the definition of *R*_*j*_; in a full cohort study *R*_*j*_ would be replaced by the full risk set at time *t*_*j*_. Tied survival times can be handled using Breslow’s method (1972) [14]. The expression in (4) is a pseudo-partial likelihood due to the ‘shared’ control group and Sandwich estimators, or an appropriate equivalent, are required to obtain correct standard errors [4]. The case-cohort analysis can be performed using standard software for Cox regression after making a small modification to the data (the entry time (start of follow-up) for cases not in the subcohort is set to be just an instant before they become a case, ensuring that these cases only appear in the denominator of the pseudo-partial likelihood at the time at which they are the case) and using robust standard errors.

We propose that a standard case-control study may be viewed as a case-cohort study under the assumption that the cases are rare in the underlying population, and assuming that the case event times are known. In a usual case-cohort study the subcohort may contain some cases by chance. However, in our situation of a standard case-control study the controls are selected from those who did not become cases during the follow-up period. If the cases are rare in the population then the controls will be approximately representative of the population in which the cases arose. Therefore, the case-control study can be viewed as a case-cohort sample with the control group forming the subcohort. The analysis is as outlined above, with controls considered to be ‘at risk’ up until their index time (the date of interview in our motivating example).

The case-cohort approach makes full use of the data by allowing controls to contribute information to all sampled risk sets *R*_*j*_ up to their index time. A particular advantage of this approach is that it allows modelling of time-varying exposure-outcome associations as a continuous function of time; that is, we are not restricted to estimating the association within time periods. However, estimating a separate HR within a series of time periods will often be a sensible analysis particularly as a starting point.

The logistic analysis described in the preceding section may be thought of as a discrete-time survival analysis. As the time periods become small and only contain a small number of cases, the appropriate analysis would be a conditional logistic regression with cases and controls in each period forming a matched set. In this case our proposed logistic analysis reusing controls across multiple time periods becomes equivalent to the case-cohort analysis.

### Extensions

#### Frequency matching of controls

Frequency matching in a standard case-control study is analogous to stratified sampling of the subcohort in a case-cohort study [15], in which the subcohort is formed of random samples from a series of strata *s* (*s* = 1, …, *S*) defined by the frequency matching criteria. In this situation the baseline hazard *h*_0_(*t*) in (3) is replaced by stratum specific baseline hazards *h*_0*s*_(*t*). The pseudo-partial likelihood in (4) is modified by replacing *R*_*j*_ by *R*_*sj*_, the set of individuals in the subcohort who are at risk at *t*_*j*_ and in the same stratum as the case which occurred at time *t*_*j*_, plus the case itself at *t*_*j*_ (if the case is not in the subcohort). Frequency matching of controls can be accommodated in the logistic analysis by replacing *δ*_0*l*_ by *δ*_0*ls*_ in (1).

#### Time-varying exposures

Many studies involve time-varying exposures. This occurs in our motivating study, in which the time scale is age and vaccination occurs at different ages, though typically before the first birthday. Focusing on a binary exposure, we let *x*(*t*) denote the exposure at time *t*, on the relevant time scale. We now separate the time scale for occurrence of the event (*t*) and the time since exposure (*u*). The case-cohort analysis accommodates both time-varying exposure-outcome associations and time-varying exposures. Under this extension the pseudo-partial likelihood is

5 $$ \prod_{j=1}^N\frac{ \exp \left(\beta \left({u}_{i_jj}\right){x}_{i_j}\left({t}_j\right)+{\gamma}^T{z}_{i_j}\right)}{{\displaystyle {\sum}_{k\in {R}_j} \exp \left(\beta \left({u}_{kj}\right){x}_k\left({t}_j\right)+{\gamma}^T{z}_k\right)}} $$

where *u*_*kj*_ denotes the time since exposure for individual *k* at event time *t*_*j*_, and *β*(*u*) models the log HR as a function of time-since-exposure. To perform analysis the data would be arranged with multiple rows per control individual to accommodate both changing exposure over time and different times since exposure; cases would still have only one row of data.

The logistic regression approach does not extend easily to accommodate a time-varying exposure with a time-varying association with the outcome. A *non-time-varying* association between a time-varying exposure and an outcome can be estimated using logistic regression by fitting separate models within a series of time periods, using current values of the time-varying exposure in each period, and pooling the estimates across periods. This gives similar results to Cox regression using time-varying exposures [16]. This approach could be extended to the setting of a time-varying exposure with a time-varying exposure-outcome association, by fitting logistic regressions within *sub-intervals* of each time period of interest for the time-varying association and obtaining a pooled estimate across sub-intervals within each time period, using current values of the time-varying exposure in each regression. However, this is cumbersome and requires sufficient numbers of cases and controls within sub-intervals. Therefore we consider the logistic regression approach to be impractical for time-varying exposures with a time-varying association with the outcome.

#### Left truncation

In the motivating study the cases were cases of TB occurring between 2003 and 2012, resulting in left truncation prior to 2003. Left truncation is accommodated in the case-cohort analysis by having control individuals enter the risk set starting only at the time from which they would have been eligible to become a case. Left truncation can be accommodated in the logistic approach by extending the definition of a control within a given time period. We propose that an individual can appear as a control in any time period in which they are observed for any length of time, including when they do not enter the risk set until part-way through the time period due to left truncation.

## Results

We use a simulation study based on the motivating example to investigate the performance of our proposed methods.

### Simulating the data

We first generated full cohort data within which cases occur in time, and then obtained a frequency matched case-control sample within that.

Full cohort data were generated for *n*_*b*_ individuals in five birth cohorts (*b* = 1, 2, 3, 4, 5) covering the period 1984–2012. Dates of birth were generated uniformly within each birth cohort. The sizes of the birth cohorts (*n*_1_, …, *n*_5_) were chosen to give particular numbers of cases in different age groups (mimicking the numbers expected in the motivating example), resulting in approximately 582 cases in each full cohort.

The exposure (vaccination status) was generated randomly from a binomial distribution within each birth cohort, using the following exposure percentages which mimic changes in vaccination uptake in the target population over time: birth cohorts 1 and 2: 60 %; birth cohort 3: 80 %; birth cohorts 4 and 5: 90 %.

We assumed the vaccine efficacy declined over time, with HR 0.25 in the time period up to 5 years after exposure, and a subsequent increase in the HR by 35 % every 5 years, giving the HRs across years since exposure periods (which here are the same as age groups): age 0–4: 0.25 , age 5–9: 0.34, age 10–14: 0.46, age 15–29: 0.62, age > =20: 0.83.

Event times were generated using a piecewise exponential model with event rates differing by 5-year age group and using the above HRs. Estimates of age-specific TB rates for the target population were obtained from 5-year average TB rates in England [17]. The number of cases per 100,000 across age groups were: age 0–4: 13, age 5–9: 14, age 10–14: 16, age 15–19: 36, age 20–24: 40, age 25–29: 45.

All individuals were followed up to the end of 2013. The index time for cases was their age at TB diagnosis and that for non-cases was their age at the end of 2013. Left truncation was introduced so that events were only observed from 2003. Cases were all individuals having the event before the censoring time at the end of 2013 and aged 19 or under at the time of becoming a case. Individuals eligible as controls in the case-control sample were those who had not had TB by the end of 2013. Controls were sampled randomly within the 5 birth cohorts such that the number of controls in each birth cohort group was the same as the number of cases, as in frequency matching. The case-control study comprises all cases plus the sampled controls.

We generated 1000 simulated case-control data sets.

### Methods

In each simulated case-control data set we estimated the exposure-outcome association within 5-year age groups 0–4, 5–9, 10–14, and 15–19, using the methods outlined below.

1. Logistic regression analysis using controls across multiple time periods.

In the logistic analyses we allow a separate intercept parameter in each birth cohort. We consider four ways of defining a control in time period *l*, which has lower limit *τ*_*lA*_ and upper limit *τ*_*lB*_, where *T*_*E*_ denotes the entry time for a given control (i.e. start of follow-up, accounting for left truncation) and *T*_*I*_ denotes the index time:

Control definition (i) *T*_*E*_ < *τ*_*lB*_, *T*_*I*_ ≥ *τ*_*lA*_. This is our proposed approach. A control individual can serve as a control in any time period in which their start of follow-up (entry time) is before the upper limit of the time period and in which their index time is after the lower limit of the time period.

Control definition (ii) *T*_*E*_ < *τ*_*lB*_, *T*_*I*_ > *τ*_*lB*_. A control individual can serve as a control in any time period in which the start of follow-up (entry time) is before the upper limit of the time period and in which their index time is after the upper limit of the time period.

Control definition (iii) *T*_*E*_ ≤ *τ*_*lA*_, *T*_*I*_ ≥ *τ*_*lA*_. A control individual can serve as a control in any time period in which their start of follow-up (entry time) is before the lower limit of the time period and in which their index time is after the lower limit of the time period.

Control definition (iv) *T*_*E*_ ≤ *τ*_*lA*_, *T*_*I*_ > *τ*_*lB*_. A control individual can serve as a control in any time period in which their start of follow-up (entry time) is before the lower limit of the time period and in which their index time is after the upper limit of the time period. This is the most stringent control definition.

2. Logistic regression analysis using each control in only one time period.

We consider an analysis in which control individuals are only used in one time period. Controls were allocated to a time period from those in which they were eligible to be a control (according to definition (i)) so as to achieve as far as possible an equal number of controls in each time period.

3. Case-cohort analysis.

The case-cohort analysis was applied allowing a separate baseline hazard within each birth cohort.

The analyses were applied in the 1000 simulated data sets. The case-cohort analysis gives estimates of HRs, while the logistic regression analysis gives ORs. Given cases are rare in the population we expect HRs and ORs to be very similar. Results are shown in Table 1.

**Table 1:** Simulation study results

	True HR	True log HR	OR or HR	Log OR or Log HR	Difference from true log HR	Emp SD	Model SE	Cov	RE
1. Logistic regression analysis using controls in multiple time periods, controls definition (i)									
Age 0-4	0.25	−1.386	0.246	−1.403	−0.017	0.260	0.258	0.943	-
Age 5-9	0.34	−1.079	0.335	−1.093	−0.015	0.238	0.244	0.962	-
Age 10–14	0.46	−0.777	0.458	−0.781	−0.004	0.235	0.234	0.947	-
Age 15-19	0.62	−0.478	0.619	−0.480	−0.002	0.205	0.197	0.943	-
1. Logistic regression analysis using controls in multiple time periods, controls definition (ii)									
Age 0-4	0.25	−1.386	0.244	−1.412	−0.026	0.290	0.293	0.947	80
Age 5-9	0.34	−1.079	0.332	−1.104	−0.025	0.267	0.270	0.952	79
Age 10–14	0.46	−0.777	0.456	−0.785	−0.008	0.260	0.257	0.948	82
Age 15-19	0.62	−0.478	0.617	−0.483	−0.005	0.230	0.226	0.940	79
1. Logistic regression analysis using controls in multiple time periods, controls definition (iii)									
Age 0-4	0.25	−1.386	0.246	−1.403	−0.017	0.301	0.293	0.948	75
Age 5-9	0.34	−1.079	0.334	−1.098	−0.020	0.260	0.266	0.954	84
Age 10–14	0.46	−0.777	0.460	−0.776	0.000	0.246	0.246	0.954	91
Age 15-19	0.62	−0.478	0.619	−0.480	−0.002	0.217	0.211	0.942	89
1. Logistic regression analysis using controls in multiple time periods, controls definition (iv)									
Age 0-4	0.25	−1.386	0.243	−1.416	−0.030	0.351	0.346	0.951	55
Age 5-9	0.34	−1.079	0.328	−1.114	−0.035	0.299	0.301	0.953	63
Age 10–14	0.46	−0.777	0.458	−0.780	−0.003	0.273	0.273	0.952	74
Age 15-19	0.62	−0.478	0.616	−0.484	−0.006	0.248	0.247	0.951	68
2. Logistic regression analysis, not using controls across multiple time periods									
Age 0-4	0.25	−1.386	0.244	−1.411	−0.025	0.318	0.307	0.954	67
Age 5-9	0.34	−1.079	0.327	−1.118	−0.039	0.317	0.315	0.946	56
Age 10–14	0.46	−0.777	0.454	−0.789	−0.012	0.305	0.301	0.950	59
Age 15-19	0.62	−0.478	0.616	−0.485	−0.007	0.218	0.214	0.939	88
3. Case-cohort analysis									
Age 0-4	0.25	−1.386	0.249	−1.390	−0.004	0.277	0.267	0.944	-
Age 5-9	0.34	−1.079	0.337	−1.087	−0.008	0.240	0.245	0.957	-
Age 10–14	0.46	−0.777	0.461	−0.775	0.002	0.236	0.233	0.942	-
Age 15-19	0.62	−0.478	0.623	−0.474	0.004	0.206	0.198	0.939	-

OR or HR: Exponential of the mean estimated log OR (logistic analyses) or log HR (case-cohort analysis) across 1000 simulations.

Log OR or log HR: Mean of the estimated log OR (logistic analyses) or log HR (case-cohort analysis) across 1000 simulations.

Difference from true log HR: Mean difference between the estimate of the log HR or log OR and the true log HR across the 1000 simulations.

Emp SD: Empirical standard deviation of the estimates of the log HRs or log ORs across the 1000 simulations.

Model SE: The mean of the model-based standard errors for the estimates of the log HRs or log ORs across the 1000 simulations.

Cov (Coverage): The proportion of the 1000 95 % confidence intervals for each of the log HRs or log ORs ratios which contain the true log HR.

RE (Relative efficiency): percentage efficiency relative to the logistic analysis using controls definition (i). The relative efficiency is the ratio of the squared empirical standard deviation for the reference method (i) to the squared empirical standard deviation for the comparison method (control definitions (ii), (iii), (iv), and not reusing controls), expressed as a percentage

### Simulation results

All analyses give estimates of the exposure-outcome association within time periods which are very close to the true HRs. The case-cohort analysis gives the estimates closest to the true HRs. All methods also give correctly estimated standard errors (comparing the empirical standard deviation with the model standard error) and good coverage.

The case-cohort approach and the logistic approach using our proposed control definition (i) gave similar precision (looking at the empirical standard deviations). The precision of the logistic regression estimates varied according the control definition and whether controls were reused across time periods. Our proposed logistic regression approach which reuses controls according to definition (i) was the most efficient. Using control definition (ii) results in around a 20 % loss in efficiency compared to definition (i). Control definition (iv) is the most stringent and gives the largest standard errors. The logistic regression approach not reusing controls across time periods also gives a substantial loss of efficiency relative to our proposed method.

## Discussion

We have outlined two approaches for estimation of time-varying exposure-outcome associations using case-control data; a logistic regression approach and a case-cohort analysis. Our simulations showed that both methods give correct estimates of the time-varying association. The methods can be used to estimate time-varying associations from case-control data in settings where this may not previously have been considered a viable study design, notably in studies of vaccine efficacy over time. The approaches outlined assume that cases are rare in the underlying population.

The case-cohort approach has a number of advantages and this is our recommended method of analysis. A major drawback of the logistic regression approach is that it is restricted to assuming a step function form for the time-varying association, i.e. estimation of the association within a series of time periods, while the case-cohort analysis accommodates a flexible model for the time-varying association.

We showed how controls can be reused across time periods in the logistic regression approach. However, a further drawback of the logistic approach is that there is ambiguity over what the definition of a control should be in a given time period. In the simulation study we considered four definitions for controls, which determine whether a control individual is eligible to contribute to the logistic regression analysis in a given time period. Our results showed that there are considerable gains in efficiency by reusing controls across time periods, and that our proposed control definition (i) is most efficient. By contrast, the case-cohort analysis automatically makes efficient use of controls and there is no ambiguity over the definition of a control at any time point, as a control individual contributes to the sampled risk set at all event times at which they were at risk. We found similar results using the case-cohort analysis and the logistic regression analysis which makes most efficient use of controls.

In summary, the case-cohort approach has several advantages over the logistic regression approach. It allows a flexible model for the time-varying exposure-outcome association and, because it handles time continuously, involves no ambiguity over the definition of a control at a given time point. Additionally, the case-cohort approach easily accommodates time-varying exposures, whereas it is impractical to do this using logistic regressions.

We have focused on unmatched and frequency matched studies. Individual matching of cases to controls is also common, including the use of matching on ‘time’ using ‘concurrent sampling’; for example matched controls are selected from those who have reached the same age as the case at his/her event time. When the matching is in continuous time, this is equivalent to a nested case-control study in which controls are sampled from the risk set for each case. In this situation, the modified partial likelihood analysis used for nested case-control data is identical to a conditional logistic regression analysis. Niccolai et al. (2007) [18] discussed the use of a nested case-control design to study vaccine efficacy over time, and Vasquez et al. (2004) [19] used a study of this type to investigate the efficacy over time of the varicella vaccine.

Use of time-varying exposures in case-control studies has been considered previously in work which is closely connected to ours. Suissa et al. (2010) [20] described a ‘multitime case-control design’ for estimating the associations between time-varying exposures and an outcome using an unmatched case-control study, motivated by transient exposures. They noted that controls could provide exposure information for multiple time periods and outlined simple approaches to estimation of ORs, though did not extend to regression modelling. The methods described in this paper are an extension of their methods to a more general setting. Leffondre et al. (2003) [21] considered use of time-varying exposures in matched case-control studies. They investigated analyses based on both logistic and Cox regression. Their ‘augmented Cox approach’ is similar to our case-cohort approach, as is the approach which was taken by Freedman et al. (2009) [22] to study the association between time-dependent information on smoking and risk of Warthin’s tumour using data from a matched case-control study. Leffondre et al. (2010) [23] extended to situations in which cases are not rare in the underlying population, by considering weighted Cox models using information on event occurrence in the underlying population. Our methods could be extended in a similar way and this is an area for future work.

## Conclusions

By using the case-cohort analysis outlined in this paper, case-control studies can be used to estimate time-varying associations in settings where they may not previously have been considered a viable study design. A logistic regression approach can also be used to estimate time-varying associations but is restricted to modelling the time-varying association using a step function and controls should be defined using our definition (i) to avoid loss of efficiency.

## Abbreviations

BCG: Bacillus Calmette-Guérin
HR: Hazard ratio
OR: Odds ratio
TB: Tuberculosis
